# Design and Test of Embedded Reconfigurable Mode Converter Based on Spontaneous Deformable Materials

**DOI:** 10.3390/ma16196420

**Published:** 2023-09-27

**Authors:** Shixiong Wang, Yilin Zhang, Jianjia Yi

**Affiliations:** 1 School of Information and Communications Engineering, Xi’an Jiaotong University, Xi’an 710049, China; sxwang@stu.xjtu.edu.cn; 2 The 10th Research Institute of China Electronics Technology Group Corporation, Chengdu 610036, China; yilinzhang@stu.xidian.edu.cn

**Keywords:** mode converter, refractive index perturbation, deformable all-dielectric material

## Abstract

The mode converter, as a passive mode conversion device in transmission lines, is well-investigated and widely implemented in various electromagnetic systems. However, most traditional mode converters can only realize a single conversion mode. Thus, a mode converter achieving multiple controllable output modes is urgently needed. In this paper, a reconfigurable mode converter operating in the microwave range is achieved by embedding a deformable all-dielectric material with quadrilateral shape into a rectangular waveguide based on coupled-mode theory. It can achieve different target modes with controllable output for the same input by exciting the deformable all-dielectric material. The design principle of the mode converter is expounded concretely and simulation is carried out using HFSS software 2022 R2. Experimental results, consisting of the simulation results, demonstrate that the proposed mode converter can achieve various mode conversions with mode purity higher than 95%. This article innovatively applies deformable materials to waveguide mode conversion, expanding the application of deformable memory materials in electromagnetic devices.

## 1. Introduction

Waveguide mode refers to the type of electromagnetic (EM) field that can propagate in a waveguide. Practically, an EM system requires various waveguide modes as input, such as electron cyclotron resonance heating [1], radar [2], antenna [3] and various measurement equipment [4,5]. Therefore, converting the waveguide mode into a transmission line is a necessary technique in the transmission, measurement, and emission of EM waves. As a typical microwave transmission line, the rectangular waveguide has been widely used in diverse applications, such as radars [6], filters [7], remote sensing equipment [8], and electronic countermeasures systems due to its characteristics of low energy cost and large power capacity [9,10,11]. Because of its fascinating properties and numerous potential applications, most works focus on designing efficient mode converter embedded in rectangular waveguides.

There are three common approaches for mode conversion: phase matching [12,13,14,15], beam shaping [16,17], and coherent interference [18,19,20]. The most popular method to achieve mode conversion in the microwave range is beam shaping, which adopts special waveguide structures to achieve mode conversion including right-angle structure [21] and hyperbolic structure [22]. The rectangular mode converters proposed by Shu [23] based on the coupling apertures were studied to realize the mode conversion in the cold test from a horizontal port to a vertical port. In the work, the rectangular septa were introduced to enhance the mode conversion efficiency and ensure an extendibility to higher-order modes. In addition, bend shape is a common structure. The first analysis for bends in waveguide is found in Reference [24]. Since then, mode conversion by precise and effective waveguide bending has been extensively studied thanks to the high-power capacity and simple structure. A TE20 mode converter based on an H-plane T-junction power dividing network was designed and measured in this paper [25]. To decrease the port reflection caused by the T-junction discontinuity, a septum and two symmetric irises were introduced in the converter, which increases the transmission efficiency to 95% at the H-band. However its shortcomings include non-coaxial input and output and high demand for manufacturing precision. As a result, other waveguide structures have been proposed for high-efficiency mode conversion. The work in Reference [26] shows a structure that rotates the connecting port by 90° to achieve the mode conversion between TE01 and TE20 modes with high efficiency. Xu [27] designed a rectangular waveguide mode converter that operates at the Ka-band based on the broadband characteristic of ridged twist waveguide and the polarization direction torsion of EM wave, which realized the conversion between TE10 and TE20 modes. 

Another approach involves using photonic crystals to control EM waves in the waveguide. The tapered-slot mode converter and the photonic crystal structure mentioned in References [28,29] are examples of this approach. The tapered-slot mode converter facilitates the transition of electromagnetic waves to a larger-scale mode in a terahertz silicon photonic-crystal waveguide, enabling real-time error-free data transmission and wireless transmission of uncompressed 4K high-definition video. The photonic crystal structure proposed in Reference [13] achieves TEM-TE11 mode conversion, which is a rare application of this technique in microwaves. However, coherent interference methods have the disadvantages of a large device footprint, limited mode conversion options, and relatively large insertion loss compared to rectangular waveguide mode converters.

In the field of mode conversion, one common approach is to introduce a perturbation in the refractive index of the waveguide along with controlling the propagation directions. This can be achieved by etching gaps in the waveguide, which is the most popular method for realizing mode conversion. There have been various designs and structures proposed for mode converters in different frequency ranges. For example, Ohana [30] designed a silicon-based waveguide mode converter in the near-infrared band using periodic etching on the silicon waveguide to achieve a energy exchange between modes. Another work [31] reported a low-loss multi-mode converter using a quasi-two-dimensional metastructure on a silicon waveguide to convert different input modes into different output modes. In addition to etching, other techniques have also been explored. Huang [14] introduced refractive index perturbations in silicon-based waveguides to achieve conversion between different modes. They found that etching on the waveguide simplifies the mode converter structure. On the theoretical side, TE-polarized mode converters [32] based on deeply etched polygonal slots have been reported, which are based on the analysis of transformation optics [33,34]. However, different polygonal slots are required for scaling the design to different mode-order converters. Researcher [35] has also proposed alternative approaches. For example, one study proposed an on-chip mode converter that achieves forward conversion using two cascaded Bragg reflection processes instead of a general backward conversion process. This converter has a tunable bandwidth and central frequency according to theoretical analysis. Another work [36] introduced a phase gradient metasurface to achieve mode conversion and asymmetric transmission of the waveguide. However, most of these mode converters operate in the infrared or near-infrared range. There is a need for mode converters in the microwave range. By applying the phase matching technique to the microwave range, it may be possible to solve the current problems to realize microwave mode converters. This would provide a simple and convenient solution for the integration of microwave range mode conversion systems.

**Figure 1:**
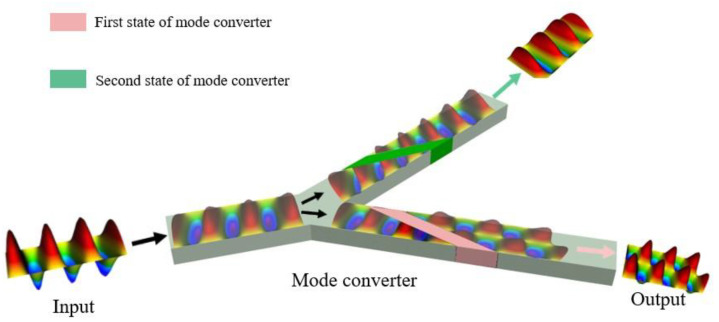
3D schematic diagram of the proposed mode converter.

To tackle the need for multiple controllable output modes in waveguide mode converters, we present a phase-matching technique utilizing an embedding reconfigurable waveguide mode converter in this work. The mode converter is designed to alter the refractive index of the standard rectangular waveguide, achieving high-purity mode conversion. Additionally, as shown in Figure 1, it has the potential to achieve one-to-many mode conversion by utilizing temperature-controlled deformable materials that introduce varying refractive index disturbances. The waveguide converter filled with red dielectric in the Figure 1 corresponds to the function of TE10-TE20 mode conversion. The time-domain solver of commercial ANSYS HFSS software 2022 R2.is used for numerical simulation to validate the design. The simulation uses national waveguide sizes WR-90 and WR-159 ports and connects matching loads at the output port. Our simulation and experimental results demonstrate that our proposed mode converter based on shape memory materials (SMMs) can achieve mode switching of TE10-TE20 and TE10-TE20 with a mode purity greater than 95%. The rectangular waveguide design is highly robust and can tolerate manufacturing defects. With SMMs under temperature excitation, the structure can be quickly and accurately reshaped to achieve mode conversion of TE10-TE30 with a purity greater than 85%. The design possesses reconfigurable performance for high-order mode conversion potential. Furthermore, the input and output port’s shape remains unaltered, providing reversibility of input-output for the waveguide converter. While achieving the TE10-TE20 conversion function, TE20-TE10 mode conversion can also occur when the waveguide is reversed. Based on 3D printing technology, the proposed mode converter can achieve different waveguide mode conversions through spontaneous deformation using temperature-controlled materials instead of altering the device’s structure, providing higher degrees of freedom for microwave section systems integration.

## 2. Methods

### 2.1. Concept, Theory and Design of The Proposed Mode Converter

According to the coupled-mode theory, if a perturbation is introduced into a waveguide, one waveguide mode can be coupled into another mode. Assuming the dielectric constant of the medium filled in the original waveguide is $\varepsilon_{a}(x,y)$, the dielectric constant after introducing perturbation can be expressed as:

(1) $$\varepsilon (x,y,z)=\varepsilon_{a}(x,y)+\Delta \varepsilon (x,y,z)$$

In the absence of perturbation, degenerate modes remain constant along the propagation direction z, making it possible to segregate the mode field distribution in terms of (x,y) from the propagation constant in the opposite direction of z. Consequently, the overall EM field distribution propagating within this waveguide can be viewed as a linear combination of all these degenerate modes:

(2) $$E=\sum_{m}A_{m}E_{m}(x,y)e^{i(\omega t-\beta_{m}z)}$$

The amplitude of each mode along the propagation direction is determined by [30]:

(3) $$\frac{d}{dz}A_{m}(z)=-\sum_{n}k_{mn}A_{m}(z)e^{i(\beta_{m}-\beta_{n})z}$$

Mode conversion is essentially the co-directional coupling of two modes in the waveguide. When considering the co-directional coupling between the two modes, (3) can be rewritten as a set of differential equations:

(4) $$\frac{\partial B}{\partial z}=jk_{mn}A_{m}e^{-j(\beta_{m}-\beta_{n}-\frac{2\pi }{\sigma })z}\;\frac{\partial A}{\partial z}=jk_{nm}A_{n}e^{-j(\beta_{n}-\beta_{m}-\frac{2\pi }{\sigma })z}$$

where $A_{m}$, $A_{n}$ represent the amplitude of modes m and n. $\beta_{m}$ and $\beta_{n}$ are the propagation constants of the two modes, $k_{mn}$ and $k_{nm}$ are exchange coupling coefficients, which reflect the coupling strength between modes m and n, respectively. The mode coupling coefficient can be defined as [17]:

(5) $$k_{mn}=\frac{\omega \varepsilon_{0}}{4}\int E_{m}^{*}(x,y)\cdot \Delta \varepsilon (x,y,z)\cdot E_{n}(x,y)dxdy$$

where ω is the angular frequency and $\varepsilon_{0}$ is the permittivity of the vacuum. $E_{m}^{*}(x,y)$ and $E_{n}(x,y)$ are the electric field distributions of the two modes, respectively. The superscript ∗ represents the complex conjugate. Δε(x,y,z) is the refractive index perturbation in the waveguide.

The proposed device functions as a co-directional coupler, facilitating the coupling of two eigenmodes that propagate in the same direction within a single multimode waveguide. Solving the mode coupling Equation (4) under the phase matching condition reveals a periodic exchange of power between the modes as they propagate [19]. The solution to the coupled mode equations is as follows:

(6) $$A_{m}(z)=\cos(sz)\cdot A_{n}(0)-i\frac{k_{mn}}{s}\sin(sz)\cdot A_{m}(0)\;A_{n}(z)=\cos(sz)\cdot A_{m}(0)-i\frac{k_{nm}^{*}}{s}\sin(sz)\cdot A_{n}(0)$$

where $s^{2}=k_{12}k_{12}^{*}+{\left(\Delta \beta /2\right)}^{2}$ and $\Delta \beta =\Delta \beta_{m}-\Delta \beta_{n}-2\pi /\sigma$. To satisfy the phase matching condition along the propagation direction, we need to compensate the mismatch (i.e., $\beta_{m}-\beta_{n}$) of the propagation constants of modes m and n to achieve maximum power transfer, which is shown in the exponential term of (6). Therefore, the phase matching condition can be deduced as follows:

(7) $$\Delta \beta =\Delta \beta_{m}-\Delta \beta_{n}-\frac{2\pi }{\sigma }=0$$

Theoretically, the period of the structure is $\sigma =2\pi /(\beta_{m}-\beta_{n})$ for periodic perturbations [17]. In this work, an aperiodic perturbation is introduced to accomplish the mode-order conversion. Followed by Reference [19], we adopt the similar relation of $L_{v}=2\pi /(\beta_{m}-\beta_{m})$ for the proposed aperiodic perturbation as the structure period for periodic perturbations, where $L_{v}$ is called the effective coupling length.

**Figure 2:**
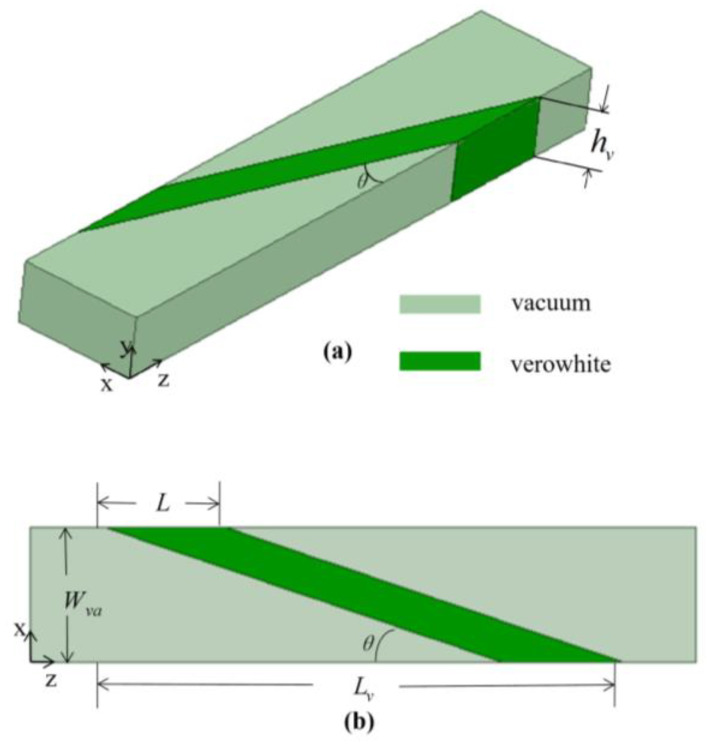
(**a**) 3D schematic diagram and (**b**) top view of the proposed mode converter.

Figure 2 shows a 3D schematic diagram of the proposed mode converter, comprising a quadrangular deformable all-dielectric material. Along the transverse (x-direction) and propagation (z-direction) directions, the quadrangular medium with angle θ and length L induces dielectric perturbations in the standard waveguide, achieving mode conversion through co-directional coupling.

### 2.2. Numerical Demonstrations of Performance

**Figure 3:**
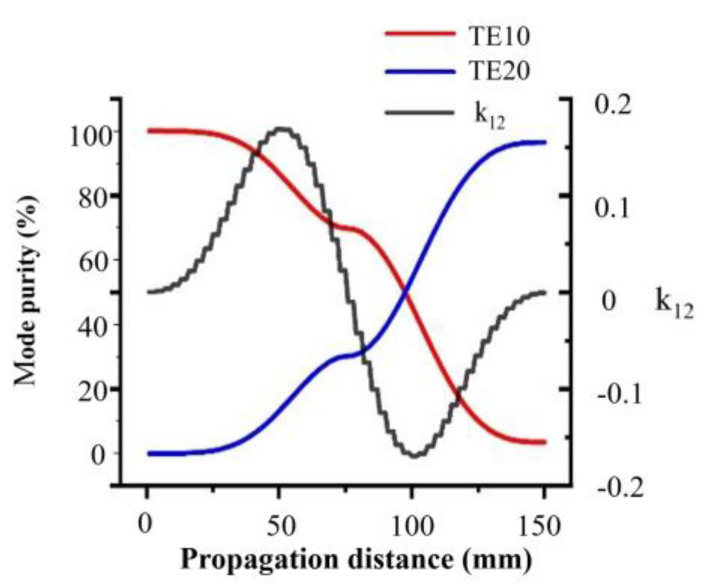
Calculated $k_{12}$ (black curve) between waveguide modes TE10 and TE20, and the mode purity of TE10 mode (red curve) and TE20 mode (blue curve) along the propagation direction z.

We obtained the approximate parameters of the coupled mode theory.$k_{01}$ between waveguide modes TE10 and TE20 and the mode purity of the two modes along the propagation direction z are shown in Figure 3. According to (6), the maximum power exchange occurs at Δβ=0 and $\left|k_{mn}\right|L_{v}=\pi /2$. The relationship of it can be calculated by:

(8) $$K_{mn}=\left|k_{mn}\right|L_{v}=\int_{0}^{L_{v}}\left|k_{mn}(z)\right|dz$$

Therefore, finding suitable parameters to satisfy (7) is the key to the design of the mode converter. In order to further improve the performance of the device, we selected an approximate interval containing parameter L, $L_{v}$ and θ to conduct multiple sets of simulation experiments.

In this work, the numerical results were obtained by ANSYS HFSS 2022 R2 based on the finite element method. In simulations, the temperature-controlled deformable material Verowhiteplus is set to a dielectric constant $\varepsilon_{r}$ = 2.8 and loss tangent tanθ = 0.02. The first step in the design process is to select a suitable width for the rectangle waveguide for supporting the converted guided modes. In the first simulation, we chose the standard waveguide WR-159 with cross-sectional parameters a = 40.386 mm and b = 20.193 mm to achieve the mode conversion. The parameters of the mode converter are shown in Table 1. Figure 3 shows the amplitude conversion between TE10 and TE20 modes of the waveguide influenced by the proposed mode converter. It can be seen that, when the medium satisfies the designed parameter, almost all the TE10 mode convert to the TE20 mode without considering the propagation loss of the medium. When the distance of electromagnetic waves passing through the medium is relatively short, and the phase changes have not yet accumulated, resulting in low mode conversion efficiency. When the electromagnetic wave completely passes through the designed dielectric structure, it can achieve the phase difference required for mode transition, and the main mode completely changes from TE10 mode to TE20 mode.

**Figure 4:**
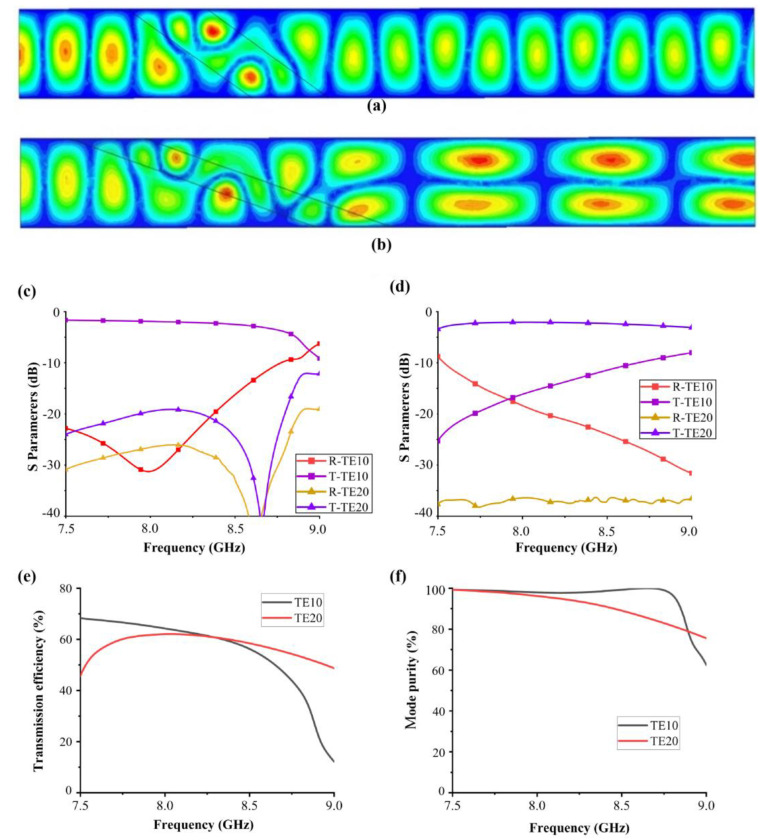
Electric field ($E_{x}$) profiles of (**a**) the first state of deformable material and (**b**) the second state of deformable material. (**c**,**d**) S parameters, (**e**) conversion efficiency and (**f**) mode purity of the two modes.

Figure 4a,b show the electric field distribution of TE10-TE10 function. It can be seen that when the inclination angle of the medium is large, although there is a certain degree of loss when EM waves pass through the medium, the main mode of propagation does not change. When the angle of the medium decreases, the phase difference intensifies, and the energy of TE10 mode couples to TE20 mode. When the medium parameters are L = 36 mm and θ = 26°, it has no influence on the propagation mode. The transmission coefficient and reflection coefficient corresponding to TE10 mode and TE20 mode are shown in Figure 4c. When the medium parameters are L = 36 mm and θ = 18.6°, the main output mode becomes TE20 mode. The transmission coefficient and reflection coefficient corresponding to TE10 mode and TE20 mode are shown in Figure 4d. Utilizing the characteristics of the embedding medium, we adjust the parameters in the same volume of the medium, which exacerbates the change in phase difference so that it can achieve different mode coupling. By utilizing the reconfigurable performance of temperature-controlled deformable materials, the proposed mode converter can achieve different output modes under the same input mode. To quantify the mode converter performance, we assess three crucial indices, including: (1) conversion efficiency-defined as the power ratio of the desired output mode to the input mode; (2) mode purity-defined as the power ratio of the desired output mode to the output mode. Figure 4e,f show the conversion efficiency and mode purity of the proposed mode converter. Due to the addition of a dielectric in the waveguide, losses are inevitable. The transmission efficiency of the mode can only reach 70%, but the mode purity is as high as 95%, proving the effectiveness of the coupled mode theory in designing dielectric waveguides.

**Figure 5:**
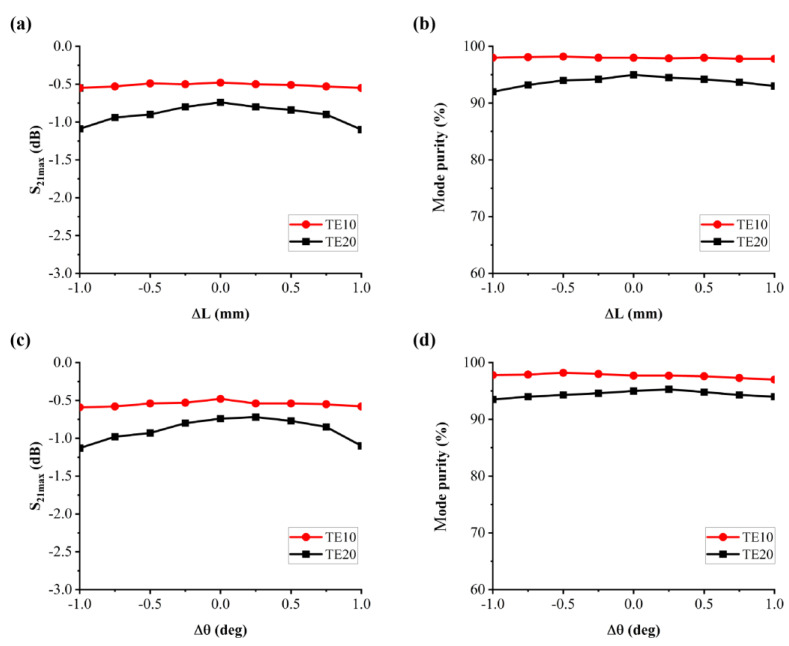
The (**a**) transmission parameter and (**b**) mode purity of the proposed mode converter within Δθ < ±1° (**c**) transmission parameter and (**d**) mode purity of the proposed mode converter within ΔL < ±1 mm of the TE10-TE20 mode converter.

To evaluate device feasibility, we investigated fabrication tolerance by examining the effects of geometry deviation on performance. We first analyzed the conversion efficiency and mode purity versus variation in angle Δθ and length of side ΔL of the embedded material, as shown in Figure 5. The transmission coefficient of the converter within Δθ < ±1° and ΔL < ±1 mm is larger than −3 dB with a mode purity of 80% and more, which indicates that the designed mode converter has good robustness.

According to the theoretical calculations, the mode converter can be applied to other waveguides to realize higher-order mode conversion. To verify the generality of the mode converter, we designed another mode converter that realizes the conversion between TE10 and TE30 modes embedded in the WR-229 waveguide with cross-sectional parameters a = 58.17 mm and b = 29.08 mm. The parameters of the mode converter are shown in Table 2.

**Figure 6:**
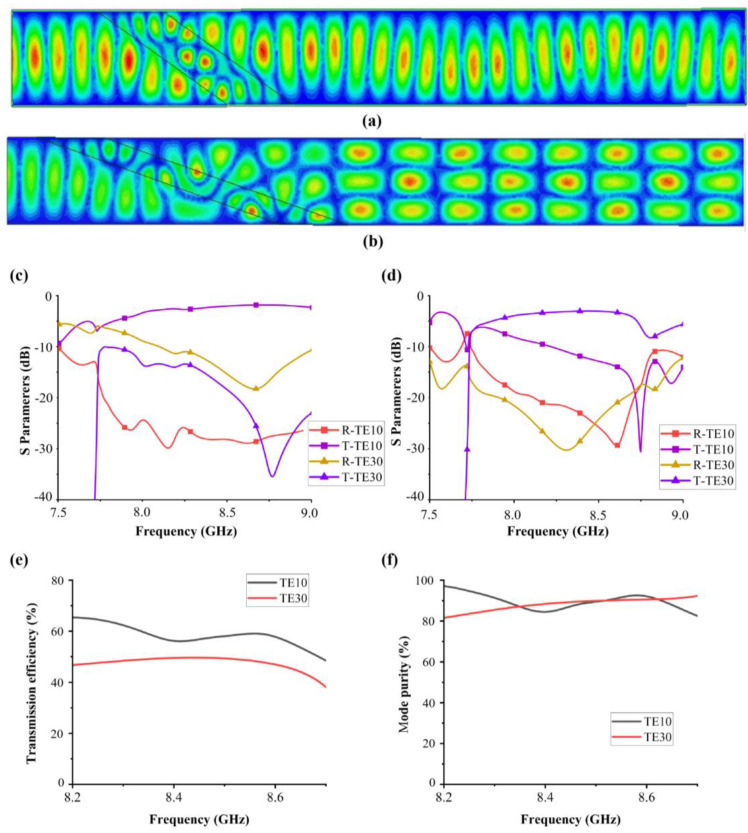
Electric field ($E_{x}$) profiles of (**a**) the first state of deformable material and (**b**) the second state of deformable material. (**c**,**d**) S parameters and (**e**) conversion efficiency and (**f**) mode purity of the two modes.

Figure 6a,b depict the electric field distribution for TE10-TE10 and TE10-TE30 mode conversion functions. Correspondingly, Figure 6c,d illustrate the transmission and reflection coefficients of these two functions. As the frequency increases, medium-induced losses intensify, leading to a reduction in mode converter efficiency. In addition, for the TE10-TE30 conversion function, the efficiency and purity of mode conversion are not as good as those of the TE10-TE20 conversion function. Another important reason is that waveguides that support high-order transmission modes also support low-order transmission modes, which can lead to a certain decrease in mode purity and transmission efficiency. However, it is worth noting that the mode purity can still reach 90%, underscoring the effectiveness of the mode converter and its potential for higher-order mode conversions, such as TE40 and TE50.

**Figure 7:**
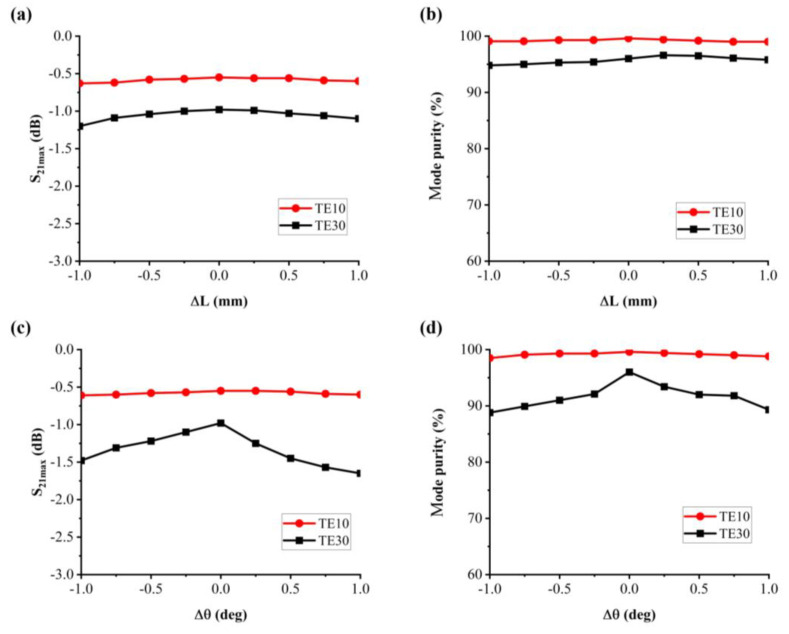
The (**a**) transmission parameter and (**b**) mode purity of the proposed mode converter within Δθ < ±1° (**c**) transmission parameter and (**d**) mode purity of the proposed mode converter within ΔL < ±1 mm of the TE10-TE30 mode converter.

To assess the robustness of the designed mode converter, we conducted an analysis of the effects of length and angle parameters on transmission efficiency and mode purity, as displayed in Figure 7. The transmission efficiency can be controlled within a range of −1.5 dB while ensuring that mode purity remains above 90%. This demonstrates that the designed device can accommodate certain processing errors and has good robustness.

## 3. Results

**Figure 8:**
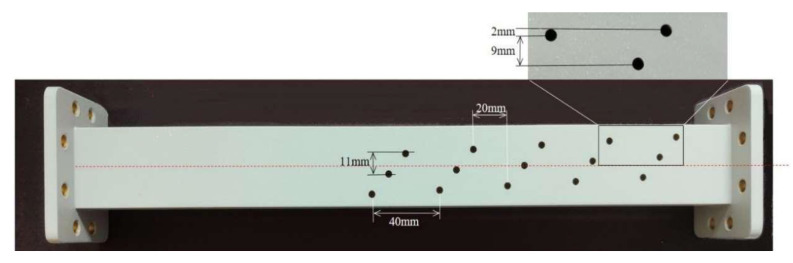
Schematic diagram of the punched standard waveguide WR-159.

To verify our simulation results and the feasibility of the proposed mode converter, we punch on the waveguide and measure the electric field amplitude propagating through the temperature-controlled deformable material to compare with the simulation results. According to the simulation results, we designed the position of the circular holes to ensure that both the high- and low-energy positions of the EM waves can be detected. The punched waveguide is shown in Figure 8.

**Figure 9:**
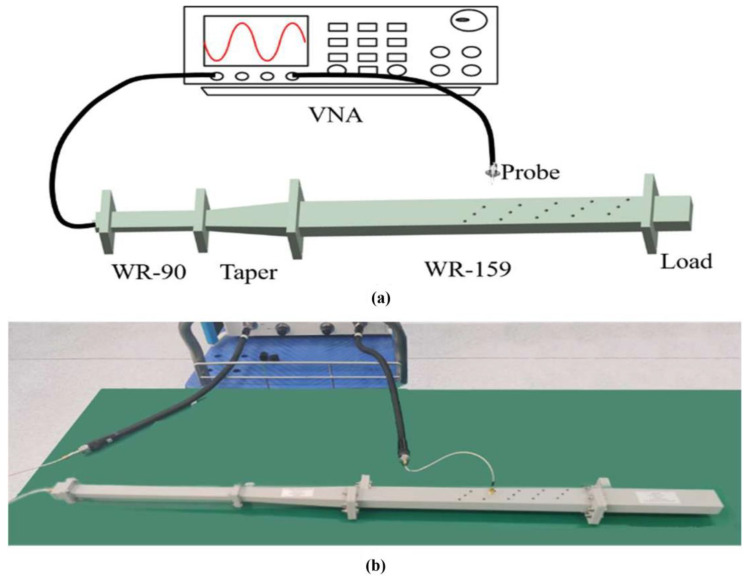
(**a**): Schematic diagram and (**b**) physical map of experimental device.

For the measurement, we employed the setup shown in Figure 9, which comprises a vector network analyzer (VNA), a dielectric probe, and the waveguide under test. To ensure that the input mode exclusively contains TE10, we used a waveguide capable of single-mode transmission at the mode converter’s operational frequency as the input, preceding the waveguide being tested. A standard WR-90 waveguide was chosen for this purpose, and a waveguide taper ensured maximum transmission of the TE10 mode into the waveguide under test. A matching load was employed to absorb both TE10 and TE20 modes. In addition, in order to minimize the interference of the inserted probe in the electric field, a dielectric probe is used in the experiment to detect the electric field amplitude at the trepanning position. We test the electric field amplitude in the two states by inserting the material at 7.8 GHz at the trepanning position, respectively. The 3D printing technology is applied to process the temperature-controlled deformable material. To ensure the shape of the temperature-controlled material change as expected after exciting, a mold is applied to fix its shape at 60 °C. In the experiment, a water bath heating method is adopted to excite the deformable material in the waveguide.

**Figure 10:**
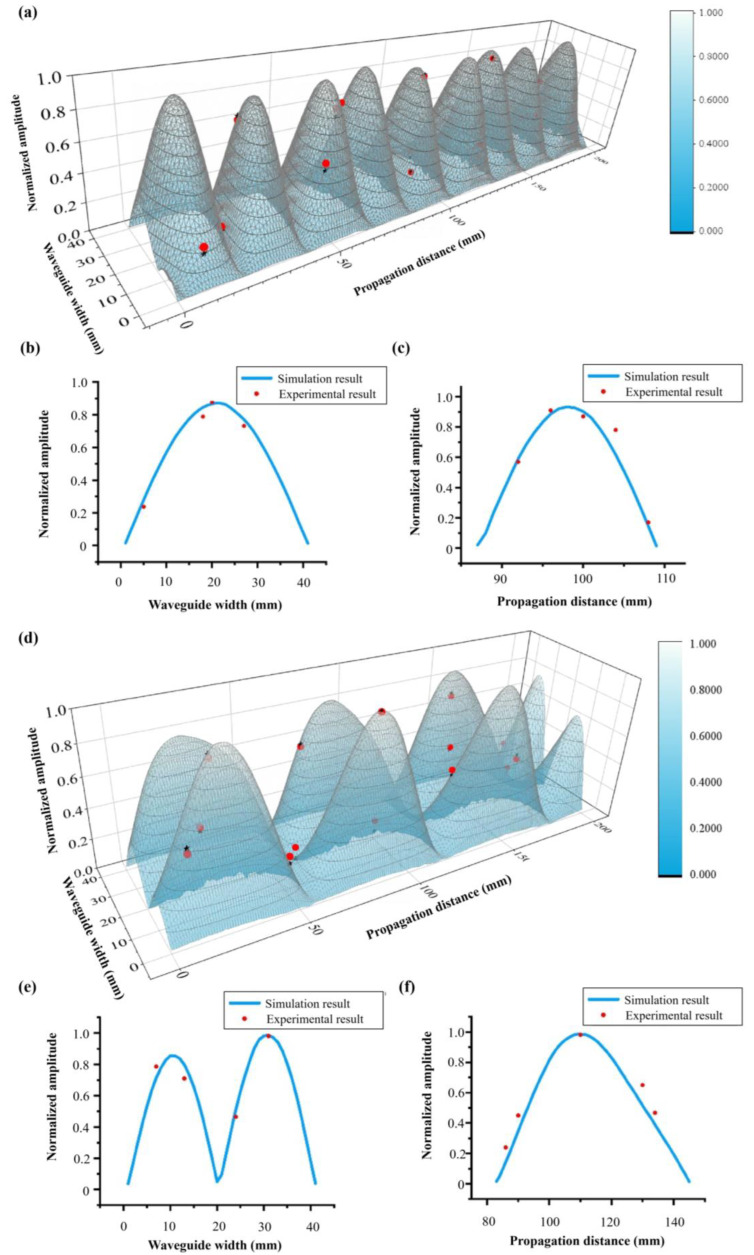
(**a**) 3D image, (**b**,**c**) sectional view along x and z direction of simulation amplitudes and experience results of electric field for the first state of the deformable material. (**d**) 3D image, (**e**,**f**) sectional view along x and z direction of simulation amplitudes and experience results of electric field for the second state of the deformable material.

We test the energy of the electric field at 7.8 GHz at the trepanning position of the waveguide with L = 36 mm and θ = 18.6°. Figure 10 presents both the simulation results and the experimental measurements. It is evident that the normalized amplitudes of the electric field at different positions, as measured by the probe, closely align with the simulation results within an acceptable margin of error. Furthermore, these results affirm that our proposed concept can be accurately extended to other frequency bands.

Table 3 compiles device-related metrics, including the relative length of the device (defined as the ratio of device length to operating wavelength), conversion efficiency, mode purity, and function for each mode converter. Here’s a breakdown of the findings:

The mode-oriented coupling/decoupling combiner proposed in Reference [13] achieves mutual conversion between the TE10 and the TE20 modes with impressive conversion efficiencies of 93% and 98.6%, respectively. However, it comes with a relatively large coupling length, approximately 32 times its working wavelength. Reference [31] introduces a periodic waveguide structure that employs waveguide etching for mode conversion, achieving a remarkable conversion efficiency of 98.8%. Despite structural optimizations, this device remains relatively lengthy, spanning 13 wavelengths. Reference [35] puts forth a mode converter based on phase gradient metasurfaces, enabling asymmetric mode transmission and conversion. While it excels in terms of device length and mode purity, it faces challenges as the number of modes increases, leading to longer coupling distances and reduced conversion efficiency. Reference [31] utilizes periodic etching to design TE10-TE20 mode conversion on silicon-based waveguides. It achieves a conversion efficiency exceeding 75% and maintains mode purity above 90%. However, its relative bandwidth is limited to only 1.35%. Reference [37] accomplishes TEp-TEq (where p < q) mode conversion through two conical waveguides and subwavelength gratings, yielding a conversion efficiency exceeding 83% and maintaining high mode purity.

In comparison to these previous works, the proposed mode converter distinguishes itself with a compact structure, robustness, exceptional mode purity, and a relatively broad bandwidth. Notably, this article innovatively leverages the characteristics of SMMs to design a mode converter that offers reversible and controllable mode output with the potential for higher-order mode conversions. However, practical applications should consider temperature variations in the waveguide environment to mitigate errors stemming from spontaneous material changes. Finally, the deformation capacity of SMMs is not infinite, and the degree of deformation of SMMs should also be considered in practical engineering applications.

## 4. Conclusions

In this work, a reconfigurable rectangular waveguide mode converter operating at the microwave range has been investigated and verified. The actual effect of the mode converter was consistent with the simulation results, which proves the effectiveness of our design theory. Based on the 3D printing technology, the proposed mode converter achieved different waveguide mode conversions by spontaneous deformation through temperature-controlled materials instead of modifying the structure of the device. Compared to other forms of mode converters in the microwave section, the mode converter proposed in this work can achieve higher purity mode conversion in a smaller size. Although this reconfigurable method is convenient and accurate, it still faces the problems of long deformation time and limited deformation degree. Moreover, numerical simulations showed that the mode converter could be extended to other frequencies by modifying the parameters of the device. The embedded features of the mode converter enable it to be applied in different kinds of rectangular waveguides. This light structure expands the application of shape memory materials to electromagnetic devices, providing an alternative to constructing novel waveguide mode converters. In the future, reconfigurable waveguide mode converters based on SMMs will definitely be applied in highly integrated microwave systems, effectively avoiding some additional operations, such as system reconstruction.

## Figures and Tables

**Table 1:** Geometry parameters of the mode converter operating at a wavelength of λ = 38.5 mm.

Function	$W_{v}$ (mm)	L (mm)	$L_{v}$ (mm)	θ
TE10→TE10	40.386	36	118.81	26°
TE10→TE20	40.386	36	157.43	18.4°

**Table 2:** Geometry parameters of the mode converter operating at a wavelength of λ = 35.3 mm.

Function	$W_{v}$ (mm)	L (mm)	$L_{v}$ (mm)	θ
TE10→TE10	58.17	36	140.94	29°
TE10→TE30	58.17	36	220.28	17°

**Table 3:** Comparison of different mode converters.

	l/λ (mm)	Conversion Efficiency (%)	Mode Purity (%)	Function
[13]	32.3	93	97	TE10-TE20
		98.8	/	TE20-TE10
[31]	3.6	95	98.7	TE10-TE20
	4.8	83	93	TE10-TE30
[31]	15	92	95	TE10-TE20
[35]	3.6	93	93.7	TE10-TE20
		82		TE10-TE30
[37]	<9.7	>83	>90	TEp-TEq
This work	4.2	84	98	TE10-TE20
	6.2	80	96	TE10-TE30

## Data Availability

Not applicable.

## References

[1] Iatrou C.T., Whaley D.R., Kern S., Thumm M., Tran M.Q., Möbius A., Nickel H.-U., Norajitra P., Wien A., Tran T.M. (1995). Feasibility study of the eu home team on a 170GHz 1MW CW gyrotron for ECH on ITER. Int. J. Infrared Millim. Waves.

[2] Danly B.G., Blank M., Calame J.P., Levush B., Nguyen K.T., Pershing D.E., Parker R.K., Felch K.L., James B.G., Borchard P. (2000). Development and testing of a high-average power, 94-GHz gyroklystron. IEEE Trans. Plasma Sci..

[3] Hosseini S.J., Oraizi H. (2020). A TEM-TE11 mode converter antenna like a pelican beak. IET Microw. Antennas Propag..

[4] Rahkala M., Suntio T., Kalliomaki K. (2002). Effects of switching frequency modulation on EMI performance of a converter using spread spectrum approach. IEEE Appl. Power Electron. Conf. Expo..

[5] Yao J., Wang S., Zhao H. (2019). Measurement techniques of common mode currents, voltages, and impedances in a flyback converter for radiated emi diagnosis. IEEE Trans. Electromagn. Compat..

[6] Salmanogli A., Gokcen D. (2021). Entanglement sustainability improvement using optoelectronic converter in quantum radar (interferometric object-sensing). IEEE Sens. J..

[7] Huang C.Y., Lin C.H., Chen Y.H., Huang Y.C. Electro-Optic Ti:PPLN Waveguides as Efficient Optical Wavelength Filters and Mode Polarization Converters. Proceedings of the Conference on Lasers and Electro-Optics.

[8] Karppanen M., Sippola M., Suntio T. Impact of remote sensing on converter stability and performance. Proceedings of the 2007 European Conference on Power Electronics & Applications.

[9] Yeddulla M., Tantawi S., Guo J., Dolgashev V. (2009). An Analytical Design and Analysis Method for a High-Power Circular to Rectangular Waveguide Mode Converter and Its Applications. IEEE Trans. Microw. Theory Tech..

[10] Tantawi S.G., Nantista C.D., Dolgashev V.A., Pearson C., Nelson J., Jobe K., Chan J., Fant K., Frisch J., Atkinson D. (2005). High-power multimode X-band rf pulse compression system for future linear colliders. Phys. Rev. Accel. Beams.

[11] Nantista C. An Alternate Dual-Moded DLDS Utilizing the TE01 and TE02 Modes. Proceedings of the XX International Linac Conference.

[12] Tantawi S.G., Nantista C.D., Dolgashev V.A., Pearson C., Nelson J., Jobe K., Chan J., Fant K., Frisch J., Atkinson D. (2013). On-chip two-mode division multiplexing using tapered directional coupler-based mode multiplexer and demultiplexer. Opt. Express.

[13] Ohana D., Levy U. (2014). Mode conversion based on dielectric metamaterial in silicon. Lasers Electro-Opt. IEEE.

[14] Hao L.J., Xiao R., Shi Y., Dai P., Zhao Y., Liu S., Lu J., Chen X. (2019). Efficient TE-Polarized Mode-Order Converter Based on High-Index-Contrast Polygonal Slot in a Silicon-on-Insulator Waveguide. IEEE Photonics J..

[15] Chen D.G., Xiao X., Wang L., Yu Y., Liu W., Yang Q. (2015). Low-loss and fabrication tolerant silicon mode-order converters based on novel compact tapers. Opt. Express.

[16] Huang Y., Xu G., Ho S.-T. (2006). An ultracompact optical mode order converter. IEEE Photonics Technol. Lett..

[17] Guan H., Ma Y., Shi R., Novack A., Tao J., Fang Q., Lim A.E.-J., Lo G.-Q., Baehr-Jones T., Hochberg M. (2014). Ultracompact silicon-on-insulator polarization rotator for polarization-diversified circuits. Opt. Lett..

[18] Liu V., Miller D.A.B., Fan S. (2012). Ultra-compact photonic crystal waveguide spatial mode converter and its connection to the optical diode effect. Opt. Express.

[19] Chen G., Kang J.U. (2005). Waveguide mode converter based on two-dimensional photonic crystals. Opt. Lett..

[20] Frandsen L.H., Elesin Y., Frellsen L.F., Mitrovic M., Ding Y., Sigmund O., Yvind K. (2014). Topology optimized mode conversion in a photonic crystal waveguide fabricated in silicon-on-insulator material. Opt. Express.

[21] Matsumoto S., Ohta I., Fukada K., Kawai T., Iio K.I., Kashiwa T. A TE10-TE20 mode transducer utilizing a right-angled corner and its application to a compact H-plane out-of-phase power divider. Proceedings of the Microwave Conference, 2009, APMC 2009, Asia Pacific IEEE.

[22] Zhang Q., Yuan C.W., Liu L. (2012). Theoretical Design and Analysis for TE20-TE10 Rectangular Waveguide Mode Converters. IEEE Trans. Microw. Theory Tech..

[23] Shu G., Cai Z., Li Y., Liu G., He W. (2020). Wideband rectangular te to ten0 mode converters for terahertz-band high-order overmoded planar slow-wave structures. IEEE Trans. Electron. Devices.

[24] Jouguet M. (1947). Effect of curvature on the propagation of electromagnetic waves in guides of circular cross sections. Cables Transm..

[25] Shu G., Qian Z., He W. (2020). Design and measurement of an h-band rectangular TE10 to TE20 mode converter. IEEE Access.

[26] Zhao P., Wang Q., Deng J. (2018). A Novel Broadband Rectangular Waveguide TE 01–TE 20 Mode Converter. IEEE Microw. Wirel. Compon. Lett. A Publ. IEEE Microw. Theory Tech. Soc..

[27] Xu Y., Peng T., Sun M., Luo Y., Wang J., Jiang W., Liu G., Wu Z. (2019). Design and test of broadband rectangular waveguide te 10 to circular waveguide te 21 and te 01 mode converters. IEEE Trans. Electron. Devices.

[28] Yu X., Kim J.Y., Fujita M., Nagatsuma T. (2019). Efficient mode converter to deep-subwavelength region with photonic-crystal waveguide platform for terahertz applications. Opt. Express.

[29] Dong W., Sha X., Yan-Wei C., Fen Q. (2014). Design of a metallic photonic crystal high power microwave mode converter. Acta Phys. Sin. -Chin. Ed..

[30] Yao C.H., Wang Z., Wang H., He Y., Zhang Y., Su Y. (2019). Multi-mode conversion via two-dimensional refractive-index perturbation on a silicon waveguide. arXiv.

[31] Huang C., Huang C. (2020). Theoretical analysis of mode conversion by refractive-index perturbation based on a single tilted slot on a silicon waveguide. Opt. Express.

[32] Leonhardt U. (2006). Optical conformal mapping. Science.

[33] Pendry J.B., Schurig D., Smith D.R. (2006). Controlling electromagnetic fields. Science.

[34] Xiao R., Shi Y., Li J., Dai P., Zhao Y., Li L., Lu J., Chen X. (2019). On-chip mode converter based on two cascaded Bragg gratings. Opt. Express.

[35] Li Z.Y., Kim M.-H., Wang C., Han Z., Shrestha S., Overvig A.C., Lu M., Stein A., Agarwal A.M., Lončar M. (2017). Controlling propagation and coupling of waveguide modes using phase-gradient metasurfaces. Nat. Nanotechnol..

[36] Dai D., Wang J., Shi Y. (2013). Silicon mode (de)multiplexer enabling high capacity photonic networks-on-chip with a single-wavelength-carrier light. Opt. Lett..

[37] Guo Z., Wu S., Xiao J. (2021). Compact and flexible mode-order converter based on mode transitions composed of asymmetric tapers and subwavelength gratings. J. Light. Technol..

